# Facial Proportions in Stunted and Non-Stunted Children Aged 7–72 Months: A Cross-Sectional Study in Bandung, Indonesia

**DOI:** 10.3390/children12081037

**Published:** 2025-08-07

**Authors:** Najwa Anindita Hidayat, Deni Sumantri Latif, Arlette Suzy Setiawan

**Affiliations:** 1 Dentistry Education Program, Faculty of Dentistry, Universitas Padjadjaran, Bandung 40132, Indonesia; najwa21012@mail.unpad.ac.id; 2 Department of Orthodontics, Faculty of Dentistry, Universitas Padjadjaran, Bandung 40132, Indonesia; deni.latif@fkg.unpad.ac.id; 3 Department of Pediatric Dentistry, Faculty of Dentistry, Universitas Padjadjaran, Bandung 40132, Indonesia

**Keywords:** facial proportions, stunting, anthropometry, craniofacial growth, children, community health

## Abstract

**Highlights:**

**What are the main findings?**

- Stunted children had significantly shorter vertical facial dimensions (N–SN and SN–M) compared to non-stunted children.
- No significant differences were observed in horizontal facial dimensions (zygomatic and intergonion width) between groups.

**What is the implication of the main finding?**

- Vertical facial measurements may serve as supportive indicators for early detection of childhood stunting.
- Facial proportion analysis offers a non-invasive and feasible approach for integration into community-based growth monitoring programs.

**Abstract:**

Stunting is a chronic growth disorder that not only affects height but may also impair craniofacial development. Facial proportions, especially in the vertical dimension, may provide additional anthropometric insight into growth status among children. **Objectives**: To assess and compare the vertical and horizontal facial proportions of stunted and non-stunted children, and to explore the potential of facial dimensions as supportive indicators for early-stunting detection in community-based settings. **Methods**: This cross-sectional analytical study involved 266 children aged 7–72 months (mean age 42.63 ± 13.82 months) from several community health centers in Bandung, Indonesia. Children were categorized as stunted or non-stunted based on WHO height-for-age Z-scores. Facial dimensions were measured directly by calibrated pediatric dentistry residents using manual instruments. The vertical dimensions included Nasion–Subnasale (N–SN) and Subnasale–Menton (SN–M), while horizontal dimensions included zygomatic width and intergonion width. Data were analyzed using the Mann–Whitney U test and Spearman correlation. **Results**: Significant differences were found in vertical facial dimensions between stunted and non-stunted children: median N–SN (32.4 mm vs. 33.6 mm; *p* = 0.003) and SN–M (42.5 mm vs. 45.1 mm; *p* < 0.001). No significant differences were observed in horizontal dimensions. All facial parameters showed a positive correlation with age (*p* < 0.001). No significant differences were found based on sex. **Conclusions**: Stunted children exhibited shorter vertical facial dimensions compared to their non-stunted peers, while horizontal dimensions remained stable across groups. Vertical facial proportions may serve as supportive indicators in the screening and monitoring of childhood stunting. This method has potential for integration into community-based growth monitoring using simple or digital anthropometric tools.

## 1. Introduction

Stunting is a manifestation of chronic growth failure in children caused by prolonged nutritional deficiencies, recurrent infections, and inadequate stimulation during the critical period of early development. According to the World Health Organization (WHO), stunting is defined as height-for-age below −2 standard deviations from the WHO growth standard curve [1]. This condition remains a major public health issue in Indonesia, with a national prevalence of 21.6% reported in 2022 according to the Indonesian Nutritional Status Survey (SSGI) [2].

In addition to affecting linear growth, stunting also impacts overall skeletal development, including the craniofacial system. Alterations or delays in facial bone growth not only have aesthetic implications but may also affect vital functions such as respiration, mastication, and speech articulation [3,4]. Therefore, evaluating facial structure may serve as an important indicator of child development.

Birth size is one of the strongest predictors of stunting. Children born small for gestational age or with low birth weight are significantly more likely to experience impaired linear and craniofacial growth trajectories in early childhood [5]. Early childhood, especially the first 1000 days, represents a critical period for linear and craniofacial growth. Growth faltering that occurs before 24 months is often irreversible, highlighting the urgency of early detection and intervention [6]. The intergenerational cycle of malnutrition, as reflected in maternal stature, also plays a key role in stunting risk, including craniofacial underdevelopment [5].

Facial proportions are anthropometric parameters that can be used to assess facial growth objectively and non-invasively. Several studies have shown that chronic malnutrition can affect facial length, particularly in the vertical dimension, which tends to be more dynamic during early childhood growth [3,7,8,9]. Facial dimension measurement using standardized photography or manual instruments has proven to be a practical and cost-effective method, especially in young children [7,10,11].

At the molecular level, chronic undernutrition is closely associated with metabolic alterations, including deficiencies in essential amino acids, which are critical for skeletal growth and bone matrix synthesis. A landmark study by Semba et al. demonstrated that stunted children have significantly lower circulating levels of essential amino acids such as lysine, leucine, and tryptophan, suggesting a biochemical mechanism linking nutritional inadequacy with impaired linear and craniofacial development [12].

Beyond the biochemical effects of nutrient deficiency, recent evidence also points to structural impacts on cartilaginous growth centers involved in craniofacial development. Recent evidence shows that malnutrition can affect not only skeletal growth but also cartilage development. A study using ultrasound measurements demonstrated that children with primary malnutrition had significantly thicker femoral cartilage than healthy peers, possibly due to delayed endochondral ossification and skeletal maturation processes [13]. This paradoxical thickening reinforces the notion that chronic undernutrition disrupts cartilage modeling, which may have implications for long-term musculoskeletal and craniofacial outcomes. Given that cartilage growth centers, such as the nasal septum and condylar cartilage, are critical in shaping facial proportions, these findings support the biological plausibility of using facial measurements as indicators of growth disturbance.

Several studies have indicated that vertical facial dimensions are more influenced by a child’s nutritional status, as they involve the dynamic growth of the maxillary and mandibular bones throughout childhood—particularly in the region from the nasion to the menton. In contrast, transverse dimensions such as interzygomatic and intergonial widths tend to be more stable from an early age and are largely determined by genetic factors. Therefore, chronic growth disturbances such as stunting are more likely to manifest in vertical facial proportions than in facial width, making vertical measurements a more sensitive indicator of delayed craniofacial development in children [3,14,15].

In addition, maternal knowledge and attitudes play a central role in shaping children’s early oral health behaviors, which may indirectly influence craniofacial development through hygiene practices, feeding habits, and dental care-seeking behavior. Recent research highlights the need to assess and improve maternal perspectives on oral health during the early years of life, especially under age three, as mothers remain the primary source of behavioral modeling during this critical period [16].

Bandung, as an urban area with diverse socioeconomic backgrounds, faces unique challenges in achieving equitable child nutrition. Therefore, this study aims to describe the facial proportions of stunted children in Bandung and to analyze differences in facial dimensions based on nutritional status, age, and sex. The findings are expected to contribute to the development of simple and effective early-detection methods for monitoring craniofacial growth in children.

## 2. Materials and Methods

This study adopted an analytical observational approach with a cross-sectional design aimed at describing the facial proportions of stunted children aged 7–72 months in Bandung, Indonesia. The study subjects were children who underwent direct facial measurements at selected integrated health posts under the supervision of community health centers) in Bandung, as well as a subset of children who were brought to the Dental Hospital of Universitas Padjadjaran for measurement purposes.

Inclusion criteria included children aged 7–72 months who were present during data collection, had nutritional status that could be classified according to WHO standards (stunted or non-stunted), and whose parents or guardians provided informed consent. Exclusion criteria included children with congenital craniofacial anomalies, a history of facial trauma, or any history of facial surgery.

**Scheme 1:**
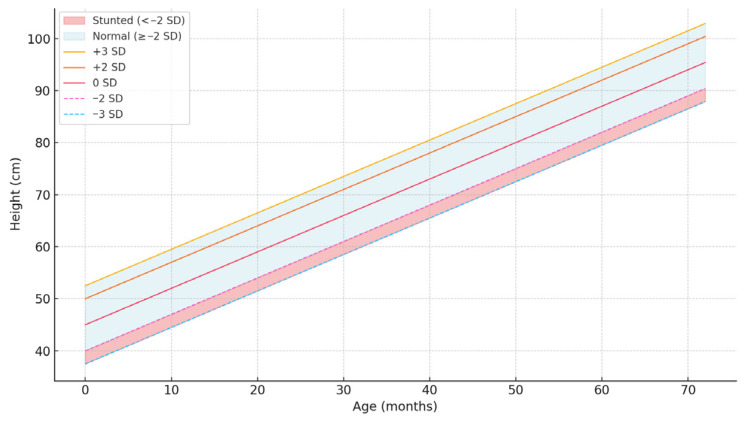
Height-for-age Z-score (HAZ) classification based on WHO standards [1].

Nutritional status was determined based on height-for-age Z-scores (HAZs), following the 2006 WHO Growth Standards. Height data were obtained from monthly measurements recorded by integrated health post cadres and community health center staff in child health books and nutritional monitoring logs. Children were classified as stunted if their Z-score was <−2SD, and as non-stunted if the Z-score was ≥−2SD (Scheme 1) [1].

**Figure 1:**
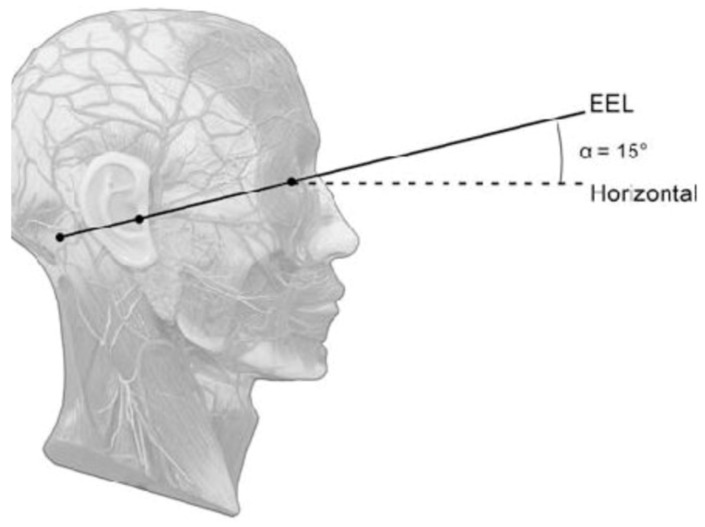
Neutral head position. The anatomic ear–eye line (EEL) is 15° to the horizontal [17].

Facial proportion measurements were performed directly using manual instruments by several residents from the Pediatric Dentistry Specialist Program. Prior to data collection, all measurers participated in technical training and a calibration session based on standardized craniofacial anthropometric protocols. Calibration was conducted by repeatedly measuring a subset of subjects and comparing measurements across instruments to ensure consistency in technique and anatomical reference points. Measurements were taken with the child’s head in a neutral head position using a sliding caliper and wide-spreading caliper, following standardized procedures to ensure inter-rater accuracy and reliability (Figure 1). Horizontal facial dimensions (interzygomatic and intergonial widths) were measured using a wide-spreading caliper and initially recorded in centimeters, and then converted to millimeters (mm) for consistency in data presentation.

The facial dimensions measured included the following:

Vertical dimensions:

- Nasion–Subnasale (N–SN): upper facial height.
- Subnasale–Menton (SN–M): lower facial height.

Horizontal dimensions:

- Zygomatic width: midfacial width (right to left zygoma).
- Intergonion width: lower jaw width (right to left gonion).

To ensure measurement reliability, intra-rater and inter-rater agreement were evaluated using intra-class correlation coefficients (ICCs). A subset of 20 children was randomly selected for repeated measurements by the same and different raters within a 1-week interval. The ICC values for vertical and horizontal facial dimensions ranged from 0.89 to 0.94, indicating excellent reliability across measurements [18,19].

Data analysis was performed using SPSS version 26. Normality was assessed using the Kolmogorov–Smirnov test. As most variables were not normally distributed, group comparisons were conducted using the Mann–Whitney U test, and correlations between age and facial dimensions were analyzed using Spearman’s rank correlation. Sex-based comparisons were also analyzed using non-parametric tests. A *p*-value of <0.05 was considered statistically significant.

This study received ethical approval from the Health Research Ethics Committee of Universitas Padjadjaran (No. 368/UN6.KEP/EC/2025). Written informed consent was obtained from the parents or legal guardians prior to participation.

## 3. Results

This study involved 266 children aged 7–72 months (mean age 42.63 ± 13.82 months), consisting of 107 stunted and 159 non-stunted children. Descriptive statistics of facial dimensions based on nutritional status are presented in Table 1.

Table 1 shows that children in the stunted group had slightly shorter vertical facial measurements than those in the non-stunted group. The median lower facial height (SN-M) in the stunted group was 42.5 mm (range: 23.5–54.7 mm), compared to 45.1 mm (29.0–57.0 mm) in the non-stunted group. Similarly, the median upper facial height (N-SN) was slightly lower in stunted children (32.4 mm) than in non-stunted children (33.6 mm).

For horizontal facial dimensions, the interzygomatic width was 80.0 mm in both groups, but with a narrower range in the stunted group (55.0–95.0 mm) versus the non-stunted group (50.0–95.0 mm). The intergonial width also showed the same median (75.0 mm), but slightly differed in range.

### 3.1. Distribution and Comparison of Facial Dimensions

Based on the Kolmogorov–Smirnov normality test on Table 1, all variables were found to be non-normally distributed (*p* < 0.05); therefore, group comparisons were performed using the Mann–Whitney U test (Table 2). The analysis showed significant differences in vertical facial dimensions between stunted and non-stunted children. The median Nasion–Subnasale (N–SN) distance in stunted children was 32.4 mm (range: 21.4–48.3 mm), lower than that of non-stunted children with a median of 33.6 mm (range: 26.3–52.3 mm) (*p* = 0.003). Similarly, the median Subnasale–Menton (SN–M) measurement in stunted children was 42.5 mm (range: 23.5–54.7 mm), compared to 45.1 mm (range: 29.0–57.0 mm) in the non-stunted group (*p* < 0.001).

In contrast, no statistically significant differences were found in the horizontal facial dimensions between the two groups (Table 2). The median interzygomatic width was identical in both groups at 80.0 mm (*p* = 0.129), and the intergonion width also showed similar medians of 75.0 cm in each group (*p* = 0.599).

### 3.2. Correlation Between Facial Dimensions and Age

Spearman correlation analysis showed that all facial parameters had a significant positive correlation with age (*p* < 0.05) in both stunted and non-stunted groups (Table 2). This indicates that as age increases, facial dimensions also tend to increase, although the rate of growth may differ between groups.

### 3.3. Differences by Sex

Further analysis revealed no significant differences in any facial parameters based on sex, in either the stunted or non-stunted groups (*p* > 0.05). The *p*-values for each dimension were 0.422 for N–SN, 0.055 for SN–M, 0.333 for interzygomatic width, and 0.167 for intergonion width (Table 3). These results indicate that sex did not influence facial dimensions within the age range studied.

## 4. Discussion

The findings of this study reinforce the understanding that nutritional status plays a critical role in craniofacial growth, particularly in the vertical dimensions of the face. The previous literature has shown that the development and elongation of facial bones are highly sensitive to chronic nutritional deficiencies, especially during the early growth period of childhood [1,20,21]. Craniofacial development is a multifactorial process, yet nutritional intervention has been proven to significantly contribute to bone differentiation and maturation [2,22].

The observed differences in vertical facial growth among stunted children may reflect these underlying metabolic disruptions. Essential amino acids play a central role in collagen formation and endochondral ossification, processes fundamental to maxillofacial development. The deficiency of these nutrients may thus contribute directly to compromised mandibular and midfacial bone growth, supporting our findings of reduced vertical dimensions in stunted subjects [12].

Prior studies have reported a strong association between vertical facial dimensions and child growth status [7,23,24]. In this context, alterations in vertical facial segments, particularly in the mandibular region, may serve as reflections of systemic growth disturbances. A study by Zhang et al. (2025) found that chronic growth restriction was associated with delayed mandibular maturation and elongation of the lower face [3]. These findings are highly relevant in the Indonesian context, where stunting remains prevalent and may affect facial structure both functionally and aesthetically.

In contrast, horizontal facial dimensions appear more stable and less affected by nutritional variations. The literature suggests that facial width is predominantly influenced by genetic factors and typically reaches maturity earlier than vertical dimensions [25,26]. Although malnutrition can impact overall growth, the body seems to prioritize the preservation of horizontal structure over vertical elongation [27].

Although our study did not assess longitudinal outcomes, previous research has shown that partial recovery from stunting is more feasible during the first five years of life [5]. This underscores the importance of early detection—such as through craniofacial indicators—to enable timely interventions.

In this study, age was positively associated with all facial parameters, indicating that facial growth continues with age even in the presence of nutritional deficits. This supports findings from a Latvian child population study, which showed that vertical facial growth increases with age, while horizontal growth remains slower and more consistent [7]. These results underscore the importance of monitoring facial development as part of comprehensive child nutritional assessments. Among stunted children, age remained positively correlated with facial dimensions, suggesting that growth persists, albeit at a potentially slower rate. Understanding this delayed growth trajectory is important, as facial development is closely linked to other functions such as speech, mastication, and respiration [8].

Our findings on facial proportions may reflect broader systemic effects of chronic undernutrition, which often co-occurs with poor sanitation, low maternal education, and limited food diversity—all of which have been identified as key determinants of stunting and growth failure [5]. In addition, craniofacial development may be influenced not only by current nutritional status but also by early-life factors such as birth weight and gestational age. Evidence indicates that low birth weight and preterm birth significantly increase the risk of stunting, which may, in turn, manifest as altered craniofacial morphology during early childhood [6].

In this context, maternal influence—particularly in shaping dietary patterns, hygiene routines, and oral health behaviors—should not be overlooked. A validated instrument developed by Suryanti and Setiawan (2021) demonstrated that maternal knowledge and attitudes toward children’s oral health can be systematically measured and may serve as an upstream indicator in child health surveillance [16]. Integrating such behavioral assessments with facial anthropometry could provide a more comprehensive understanding of risk factors for impaired craniofacial growth.

The relationship between stunting and craniofacial development may also be mediated by oral functional factors and behavioral habits, including mouth breathing, prolonged bottle feeding, and other parafunctional activities—many of which are influenced by early-life environment and parenting. Evidence from a community-based oral health empowerment program in Indonesia highlighted that nutritional insufficiency during early childhood can delay ossification centers, contributing to both skeletal growth restriction and the development of malocclusion [26]. Early interceptive orthodontic approaches have been shown to reduce the risk and severity of such developmental deviations by supporting proper dentofacial growth [28].

On the other hand, sex did not significantly affect facial dimensions in this early-age group. This finding aligns with the theory of secondary sexual development, which states that sexually dimorphic facial traits tend to emerge during puberty under hormonal influence [29]. Thus, facial morphology in children aged 7–72 (mean age 42.63 ± 13.82 months) tends to show minimal differences between boys and girls.

While this study provides strong evidence for the association between nutritional status and vertical facial growth, it is important to acknowledge potential confounding variables that were not directly measured, such as socioeconomic status, dietary patterns, and frequency of infections. These factors may independently or synergistically influence both general growth and craniofacial development. Future studies should consider adjusting for these variables to better isolate the effects of chronic malnutrition on facial proportions.

This study has several limitations. Although the data were obtained through direct measurement, manual techniques remain prone to operator variability despite prior calibration. In addition, the study focused on linear measurements of facial length and width, without accounting for facial shape or function. The relationship between facial structure and physiological functions such as breathing, chewing, or articulation was not explored. Future research should employ longitudinal designs to observe the trajectory of facial growth over time. Three-dimensional morphometric or standardized digital imaging approaches are recommended to provide a more holistic analysis. Furthermore, the integration of facial anthropometric parameters into community-based stunting screening programs may enhance early-detection strategies and support more comprehensive growth monitoring in children. Integrating craniofacial measurements into growth surveillance may address gaps in conventional monitoring systems, especially in low-resource settings where early identification of growth faltering remains a challenge [6]. Growth patterns in stunted children may differ across age groups and underlying conditions, and that longitudinal studies are needed to fully understand the trajectory of mandibular development in chronically undernourished children.

## 5. Conclusions

This study demonstrates that nutritional status, particularly stunting, influences vertical facial growth in early childhood, while horizontal facial dimensions remain relatively stable. These findings highlight that chronic growth disturbances not only affect stature, but they also reflect delayed craniofacial development, especially in the vertical axis.

The results support the potential use of facial proportions—particularly vertical measurements—as additional indicators in child growth monitoring. This approach may strengthen community-based stunting screening strategies by offering a non-invasive, rapid, and low-resource alternative to conventional anthropometric methods.

As a follow-up, it is recommended that facial dimension measurements be integrated into Growth and Development Monitoring (PPD) programs at Posyandu using simple tools or validated, standardized photo-based technology. This innovation could serve as a complementary method for early detection of growth disorders, while also contributing to the national effort in reducing stunting prevalence in a more measurable and comprehensive manner.

## Figures and Tables

**Table 1:** Descriptive statistics of facial dimensions by nutritional status.

Nutritional Status	N-SN (mm) Median (min–max)	SN-M (mm) Median (min–max)	Interzygomatic (mm) Median (min–max)	Intergonion (mm) Median (min–max)
Stunting	32.4 (21.4–48.3)	42.5(23.5–54.7)	80.0 (55.0–95.0)	75.0 (55.0–90.0)
Non-stunting	33.6 (26.3–52.3)	45.1(29.0–57.0)	80.0 (50.0–95.0)	75.0 (60.0–85.0)
Normality test (Kolmogorov–Smirnov)				
	N-SN (mm)	SN-M (mm)	Interzygomatic (mm)	Intergonion (mm)
Stunting	0.01	0.00	0.00	0.00
Non-stunting	0.00	0.00	0.00	0.00

**Table 2:** Results of Mann–Whitney U test and Spearman correlation between facial dimensions, nutritional status, and age.

Facial Parameter	Mann–Whitney U (*p*)	Significance of Nutritional Status Difference	Correlation with Age (r_s_)	Correlation *p*-Value
N-SN	0.003	Significant	0.481	<0.001
SN-M	0.000	Significant	0.401	<0.001
Interzygomatic	0.129	Non-Significant	0.317	<0.001
Intergonion	0.599	Non-Significant	0.307	<0.001

**Table 3:** Results of Mann–Whitney U test between facial dimensions and sex.

Facial Parameter	*p*-Value	Remarks
N-SN	0.422	Non-significant
SN-M	0.055	Non-significant
Interzygomatic	0.333	Non-significant
Intergonion	0.167	Non-significant

## Data Availability

The datasets generated and analyzed during the current study are available from the corresponding author upon reasonable request. Due to ethical considerations and participant confidentiality, the data are not publicly available.
